# Análise de Desfechos após Parada Cardiorrespiratória Extra-Hospitalar

**DOI:** 10.36660/abc.20230406

**Published:** 2023-07-19

**Authors:** Thais Rocha Salim, Gabriel Porto Soares

**Affiliations:** 1 Universidade Federal do Rio de Janeiro Rio de Janeiro RJ Brasil Universidade Federal do Rio de Janeiro, Rio de Janeiro, RJ – Brasil; 2 Universidade de Vassouras Rio de Janeiro RJ Brasil Universidade de Vassouras, Rio de Janeiro, RJ – Brasil

**Keywords:** Parada Cardiorrespiratória, Reanimação Cardiopulmonar, Parada Cardíaca Extra-Hospitalar

A parada cardiorrespiratória (PCR) é definida como a cessação da atividade mecânica do coração, confirmada pela ausência de sinais de circulação, clinicamente apresentados por irresponsividade, ausência de pulso e respiração ou gasping.^1^ A PCR é a via final e mecanismo de morte de diversas situações clínicas ou traumáticas que variam com a idade do paciente e local de ocorrência.^2^ A reversão da PCR e o prognóstico após o evento dependem da identificação e instituição de medidas de reanimação de alta eficácia.^3^ As taxas de Retorno à Circulação Espontânea (RCE), sobrevida até a alta hospitalar e a condição neurológica a curto e médio prazos são imprecisas e variam na literatura.^4^ Em uma metanálise que incluiu 141 estudos da América do Norte, Europa, Asia e Oceania, o RCE ocorreu em 29,7% dos pacientes adultos reanimados no ambiente extra-hospitalar e menos de 10% sobreviveram até a alta hospitalar.^5^ As maiores taxas de sobrevida ocorreram na PCR presenciada e com início das manobras de reanimação de forma precoce.^5^ Em outra metanálise, que incluiu 44 estudos realizados na Europa, Asia e América do Norte a utilização de circulação extracorpórea na reanimação extra-hospitalar apresentou taxas de sobrevida até a alta de 24%, e 18% dos pacientes apresentaram condições neurológicas favoráveis. Porém, nos estudos avaliados, o tempo para chegada do serviço médico móvel foi inferior a 5 minutos o que pode ter influenciado nos desfechos.^6^

No Brasil, no ambiente extra-hospitalar, a taxa de sobrevida está relacionada ao ritmo da PCR. Os ritmos chocáveis, taquicardia ventricular ou fibrilação ventricular, correspondem a quase 80% dos eventos e quando a desfibrilação é realizada entre 3-5 minutos do início da PCR a taxa de sobrevida fica em torno de 50% a 70%. Enquanto os ritmos não chocáveis, como atividade elétrica sem pulso e assistolia, a sobrevida é inferior a 17%. Segundo a Diretriz de Ressuscitação Cardiopulmonar e Cuidados Cardiovasculares de Emergência da Sociedade Brasileira de Cardiologia, a taxa de sobrevida nas PCRs de etiologia traumática é inferior a 3%.^1^ O artigo publicado nos Arquivos Brasileiros de Cardiologia,^7^ traz informações importantes sobre as taxas de sobrevida e condições neurológicas nas PCRs extra-hospitalares em uma capital brasileira, o que permite traçar estratégias de prevenção desse evento e aperfeiçoamento das equipes de atendimento à PCR. Trata-se de um estudo de coorte em Campo Grande, Mato Grosso do Sul (MS), Brasil. Foi realizada a coleta retrospectiva das informações das fichas de Atendimento Pré-hospitalar do serviço de atendimento móvel de urgência, de 852 vítimas maiores de 18 anos de PCR extra-hospitalar, ocorrida no período de janeiro de 2016 a dezembro de 2018. O segmento da coorte foi feito através da coleta de informações dos prontuários das unidades hospitalares em que os sobreviventes foram internados ou por entrevista com os pacientes e seus familiares.

Os autores observaram que a PCR extra-hospitalar ocorreu com maior frequência no sexo masculino, com média de idade de 64,33 anos e 70% apresentavam pelo menos uma comorbidade, sendo as mais prevalentes a hipertensão arterial, cardiopatias e diabetes. Tais resultados sinalizam a importância da prevenção e tratamento das doenças cardiovasculares para a redução de óbitos e a ocorrência das PCRs.^8 - 10^ O local mais frequente da PCR foi o domicílio (80,87%) e a etiologia da PCR foi clínica em 89,44% dos casos. O tempo de resposta médio até a chegada do primeiro atendimento foi de 13,37 (DP=7,35) minutos; até a chegada do suporte avançado de vida, foi de 19,25 (DP=10,85) minutos. Em 73,35% dos atendimentos, o primeiro ritmo detectado foi não chocável. Após a primeira PCR, 29,93% dos pacientes apresentaram RCE e 15,14% tiveram recidiva de PCR ainda no pré-hospitalar. A sobrevida até a internação hospitalar foi de 20,66%, e até a alta hospitalar de 4,23%. Dentre os sobreviventes que receberam alta hospitalar foi realizada a Escala de Categoria de Performance Cerebral,^11^ um sistema de pontuação que permite avaliar a capacidade funcional após PCR com base em entrevistas à família e em informação registrada, sendo realizada na alta, aos seis meses e em um ano. Em mais da metade dos indivíduos sobreviventes o desfecho de performance cerebral foi considerado adequado em todos os períodos de avaliação.

Apesar de o tempo de atendimento ter sido maior que o recomendado na literatura,^1 - 3^ e o primeiro ritmo detectável ter sido não chocavel, as taxas de RCE e de admissão hospitalar se igualaram ao encontrado em outros estudos na Europa e América do Norte, porém a sobrevida a alta hospitalar foi menor.^3 - 5^ Melhorar o reconhecimento e a assistência às PCRs com redução do tempo de atendimento poderia aumentar a sobrevida. A causa traumática da PCR foi associada a maior sobrevivência pré e intra- hospitalar, porém não houve diferença entre as curvas de sobrevida até a alta hospitalar. O que demonstra que as manobras de RCP devem ser executadas independentemente da etiologia da PCR e levanta a hipótese de que a qualidade do atendimento hospitalar seria um fator associado à alta.

O presente estudo tem o mérito de fornecer as características e desfechos da PCR extra-hospitalar em uma grande capital brasileira. Além, de reafirmar a importância do preenchimento adequado das fichas de atendimento pré-hospitalar. Por fim, é importante ressaltar que os resultados deste estudo podem auxiliar no planejamento de políticas de saúde e na construção das diretrizes de reanimação no país.
