# Supplementary material for: Análise de Desfechos após Parada Cardiorrespiratória Extra-Hospitalar
Source: Arq Bras Cardiol. 2023 Jul 19;120(7):e20230406. [Article in Portuguese] doi: 10.36660/abc.20230406 (PMC10382151; doi:10.36660/abc.20230406)
Supplement: Translation

# Translation: Outcome Analysis after Out-of-Hospital Cardiac Arrest

**Authors:** Thais Rocha Salim, Gabriel Porto Soares

**Affiliations:** 1 Brasil Universidade Federal do Rio de Janeiro, Rio de Janeiro, RJ – Brasil; 2 Brasil Universidade de Vassouras, Rio de Janeiro, RJ – Brasil

**Keywords:** Heart Arrest, Out-of-Hospital Cardiac Arrest, Cardiopulmonary Resuscitation

Cardiac arrest (CA) is defined as the cessation of mechanical activity of the heart, confirmed by the absence of signs of circulation, clinically manifested by unresponsiveness, absence of pulse, and breathing or gasping.^1^ CA is the final pathway and mechanism of death in various clinical or traumatic situations that vary with the patient’s age and the occurrence location.^2^ The reversal of CA and the prognosis after the event depend on identifying and implementing high-efficiency resuscitation measures.^3^ Rates of Return of Spontaneous Circulation (RSC), survival until hospital discharge, and short- and medium-term neurological condition are imprecise and vary in the literature.^4^ A meta-analysis of 141 studies from North America, Europe, Asia, and Oceania showed that RSC occurred in 29.7% of adult patients resuscitated in the out-of-hospital environment, and less than 10% survived until hospital discharge.^5^ The highest survival rates occurred in witnessed CA cases with early initiation of resuscitation maneuvers.^5^ In another meta-analysis that included 44 studies conducted in Europe, Asia, and North America, the use of extracorporeal circulation in out-of-hospital resuscitation resulted in survival rates of up to 24%, with 18% of patients having favorable neurological conditions. However, in the evaluated studies, the time for arrival of the mobile medical service was less than 5 minutes, which may have influenced the outcomes.^6^

In Brazil, in the out-of-hospital environment, survival rates are related to the rhythm of CA. Shockable rhythms, ventricular tachycardia, or ventricular fibrillation account for nearly 80% of events, and when defibrillation is performed between 3-5 minutes from the onset of CA, the survival rate is around 50% to 70%. Meanwhile, non-shockable rhythms, such as pulseless electrical activity and asystole, have a survival rate of less than 17%. According to the Guidelines for Cardiopulmonary Resuscitation and Emergency Cardiovascular Care of the *Sociedade Brasileira de Cardiologia* ,^7^ the survival rate in traumatic CA cases is less than 3%.^1^ An article published in the *Arquivos Brasileiros de Cardiologia* provides important information on survival rates and neurological conditions in out-of-hospital CAs in a Brazilian capital, allowing for the development of strategies to prevent such events and improve the *cardiopulmonary resuscitation* **(** CPR) response teams. It is a cohort study conducted in Campo Grande, Mato Grosso do Sul (MS), Brazil. Retrospective data collection was performed from the Prehospital Care records of the Mobile Emergency Care Service, involving 852 victims over 18 years old who experienced out-of-hospital CA between January 2016 and December 2018. The cohort segment was established by collecting information from the medical records of the hospital units where the survivors were admitted or through interviews with the patients and their family members.

The authors observed that out-of-hospital CA occurred more frequently in males, with a mean age of 64.33 years, and 70% had at least one comorbidity, with hypertension, heart disease, and diabetes being the most prevalent. These results highlight the importance of preventing and treating cardiovascular diseases to reduce deaths and the occurrence of CAs.^8 - 10^ The most frequent location of CA was the home (80.87%), and the etiology of CA was clinical in 89.44% of cases. The average response time until the first assistance arrived was 13.37 (SD=7.35) minutes, and until the arrival of advanced life support, it was 19.25 (SD=10.85) minutes. In 73.35% of the cases, the first detected rhythm was non-shockable. After the first CA, 29.93% of the patients achieved ROSC, and 15.14% experienced recurrent CA in the prehospital phase. The survival rate until hospital admission was 20.66%, and until hospital discharge was 4.23%. Among the survivors discharged from the hospital, the Cerebral Performance Category Scale,^11^ was used, a scoring system that evaluates functional capacity after CA based on interviews with the family and recorded information. It was conducted at discharge, six months, and one year. In more than half of the surviving individuals, the outcome of cerebral performance was considered appropriate in all evaluation periods.

Despite the longer response time than recommended in the literature^1 - 3^ and the first detectable rhythm being non-shockable, the rates of RSC and hospital admission were comparable to those found in other studies in Europe and North America. However, survival until hospital discharge was lower.^3 - 5^ Improving the recognition and assistance for CAs with reduced response time could increase survival rates. The traumatic cause of CA was associated with higher pre- and intra-hospital survival, but there was no difference in survival curves until hospital discharge. This demonstrates that CPR maneuvers should be performed regardless of the etiology of CA and raises the hypothesis that the quality of hospital care is a factor associated with discharge.

This study has the merit of providing the characteristics and outcomes of out-of-hospital CA in a major Brazilian capital. Furthermore, it reaffirms the importance of adequately completing prehospital care records. Finally, it is important to emphasize that the results of this study can assist in the planning of health policies and the development of resuscitation guidelines in the country.
